# Solvent-mediated outer-sphere CO_2_ electro-reduction mechanism over the Ag111 surface†

**DOI:** 10.1039/d1sc07119j

**Published:** 2022-02-24

**Authors:** Vivek Sinha, Elena Khramenkova, Evgeny A. Pidko

**Affiliations:** Inorganic Systems Engineering, Department of Chemical Engineering, Faculty of Applied Sciences, Delft University of Technology Delft The Netherlands v.sinha@tudelft.nl e.a.pidko@tudelft.nl

## Abstract

The electrocatalytic CO_2_ reduction reaction (CO_2_RR) is one of the key technologies of the clean energy economy. Molecular-level understanding of the CO_2_RR process is instrumental for the better design of electrodes operable at low overpotentials with high current density. The catalytic mechanism underlying the turnover and selectivity of the CO_2_RR is modulated by the nature of the electrocatalyst, as well as the electrolyte liquid, and its ionic components that form the electrical double layer (EDL). Herein we demonstrate the critical non-innocent role of the EDL for the activation and conversion of CO_2_ at a high cathodic bias for electrocatalytic conversion over a silver surface as a representative low-cost model cathode. By using a multiscale modeling approach we demonstrate that under such conditions a dense EDL is formed, which hinders the diffusion of CO_2_ towards the Ag111 electrocatalyst surface. By combining DFT calculations and *ab initio* molecular dynamics simulations we identify favorable pathways for CO_2_ reduction directly over the EDL without the need for adsorption to the catalyst surface. The dense EDL promotes homogeneous phase reduction of CO_2_*via* electron transfer from the surface to the electrolyte. Such an outer-sphere mechanism favors the formation of formate as the CO_2_RR product. The formate can undergo dehydration to CO *via* a transition state stabilized by solvated alkali cations in the EDL.

## Abstract 2

In addition to the commonly accepted inner-sphere mechanism for e^−^ transfer, we show that an outer-sphere electron transfer from the cathode to CO_2_ is operable at high overpotentials.

## Graphical abstract

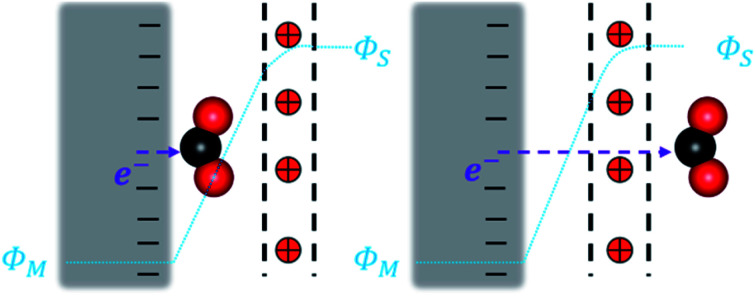

## Introduction

Electrochemical conversion of CO_2_ holds promise to help mitigate the carbon footprint of the production of fuels and chemicals.^1^ The abundant CO_2_ green-house waste gas is an attractive substrate to stabilize excess “electrons” generated from renewable energy *via* the CO_2_ reduction reaction (CO_2_RR).^2^ A wide range of electrocatalysts have been described so far for the CO_2_RR.^3,4^ Depending on the catalyst employed the primary CO_2_RR product can be either formate or CO resulting from a 2e^−^ reduction, or multi-electron transfer products such as alcohols and hydrocarbons. The electrocatalytic reduction of CO_2_ to CO opens a path for carbon recycling within the established syngas chemistry infrastructure to produce fuels and chemicals.^1^

In an electrocatalytic cell, the CO_2_RR proceeds on the cathode side. The cathode material, its morphology and electrolyte properties collectively influence the electrocatalytic activity and selectivity at the solid–liquid interface.^5,6^ Gold-based electrocatalysts have been reported to reduce CO_2_ to CO with high activity and selectivity. Hori and co-workers used bulk Au to reduce CO_2_ to CO with 87.1% faradaic efficiency (FE) at −1.14 V (NHE) with a partial CO current of 5 mA cm^−2^.^7^ At the same partial current, Ag showed a FE of 81.5% towards CO at −1.37 V (NHE).^7^ The lower cost, and comparable selectivity and activity to Au make Ag an attractive electrocatalyst for the CO_2_RR.

The selectivity and activity of an electrocatalyst for the CO_2_RR are strongly influenced by the electrolyte and the local environment close to the cathode.^5,6,8–11^ At potentials (*Φ*_M_) below the potential of zero charge (pzc) (*Φ*_pzc_) the negative charge density on the cathode surface increases, attracting more cations and resulting in the formation of an electrical double layer (EDL). The EDL influences the local electrochemical environment close to the cathode surface such as the interfacial pH and the structure of water at the interface.^11–13^ The cations in the EDL also interact with the surface intermediates and tune the stabilization of transition states and adsorbates on the electrocatalyst surface.^14,15^ However, at a higher cathodic bias, the EDL becomes very dense and compact, thus strongly hampering the mass transport of CO_2_ to the electrocatalyst surface.^16–18^

**Fig. 1:**
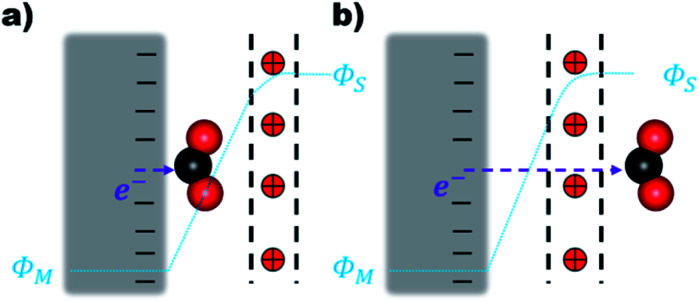
(a) Inner-sphere and (b) outer-sphere electron transfer from the cathode to CO_2_. The red positive charges within the black dotted lines denote the EDL. (*Φ*_M_) and (*Φ*_s_) are the surface and bulk solution phase potentials respectively.

The electron transfer (ET) from the cathode to the reagent is the key mechanistic step of any electrocatalytic conversion. ET can in principle proceed *via* two alternative mechanisms: inner- and outer-sphere ET (Fig. 1). The inner-sphere mechanism starts with the chemisorption of the reagent (CO_2_) to the catalyst surface that enables direct ET *via* overlapping orbitals (Fig. 1a). In the outer-sphere mechanism indirect ET from the catalyst surface to CO_2_ takes place through the electrolyte without the direct chemical interaction between the reagent and the electrocatalyst (Fig. 1b).

The inner-sphere mechanisms describing the electrocatalytic conversions in the framework of surface adsorbed species dominate the current literature.^5,19–25^ Investigating the selectivity of the CO_2_RR on various Ag facets, Bohra and co-workers proposed that the formation of formate species is self-inhibited on Ag surfaces resulting in improved selectivity to CO at low to moderate potentials, and to H_2_ at higher potentials.^26^ Their work did not consider the effects from the EDL and the electrolyte explicitly. A realistic description of the reaction medium and conditions in modelling studies has been currently emphasized across the field of catalysis.^27–40^ The importance of including an explicit representation of the EDL and accounting for the reaction conditions in mechanistic studies of the electrocatalytic CO_2_RR has been emphasized in recent literature.^14,15,41,42^

However, most mechanistic studies assume facile mass transport of CO_2_ from the bulk phase to the surface *via* the EDL. Such an assumption is reasonable for hydrodynamic transport through a low concentration electrolyte but under the operando CO_2_RR potentials, the EDL can get more condensed and strongly impact the mass transport of CO_2_.^16,17^ Under such conditions an outer-sphere ET in the homogeneous phase over the EDL is a plausible mechanism for the CO_2_RR.

Herein, we have taken a multiscale operando modelling approach to investigate the possibility and the impact of homogeneous ET on the CO_2_RR under realistic electrocatalytic conditions. The combination of classical molecular dynamics (CMD) and *ab initio* molecular dynamics (AIMD) simulations shows that CO_2_ can be favourably reduced to formate anions *via* outer-sphere ET over the dense EDL. The formate species can then convert to CO *via* a thermally activated dehydration reaction facilitated by the solvated cations within the EDL.

## Results and discussion

### Molecular structure of the EDL

**Fig. 2:**
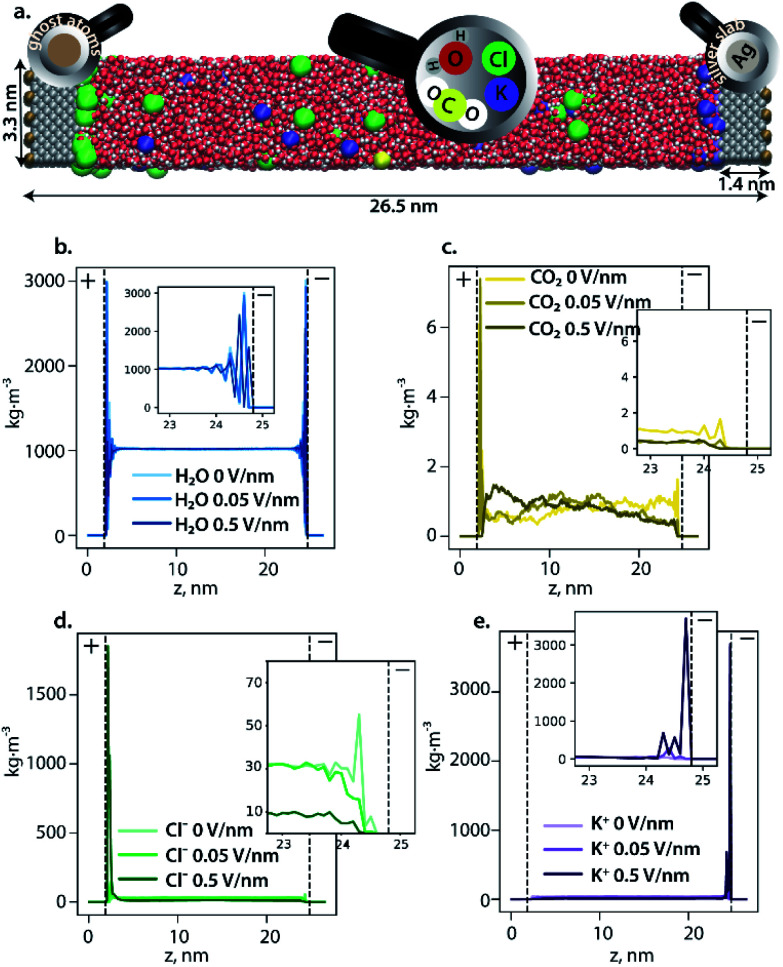
(a) A snapshot of the model of the KCl electrolyte with CO_2_ confined between two silver slabs simulated at 0.5 V nm^−1^. The surface on the left represents the anode, with positive ghost charges imposed behind the wall, while the right surface represents the cathode, with negative ghost charges imposed behind the wall. The color code is as follows: silver is grey, oxygen is red, hydrogen is white, potassium is violet, chlorine is green, carbon of CO_2_ is yellow, and the ghost atoms are light brown. (b–e) Density profile of H_2_O (b), CO_2_ (c), Cl^−^ (d), and K^+^ (e), under increasing polarization conditions represented by the electric field at the surface of the electrode. At low or zero polarization most of the ions are present in the bulk phase. With increasing polarization, the respective densities of K^+^ at the cathode and Cl^−^ at the anode show a sharp increase leading to the formation of compact EDLs at the respective electrodes.

To rationally construct an atomistic operando model of the cathode-electrolyte interface under the reaction conditions, the formation and structure of the EDL at the Ag111 surface was first investigated by classical molecular dynamics (CMD) simulations. The electrocatalytic system was modelled as an aqueous electrolyte containing 0.86 M KCl and 0.06 M CO_2_, confined between two Ag111 slabs (the model cathode and the anode) in a super cell of dimensions 33.1 × 37.2 × 265.5 Å^3^ periodic in the *x* and *y* directions (see Fig. 2a). Complete details of the CMD model and simulations are presented in the ESI.† These simulations aimed to probe the formation of the EDL at the electrodes under different polarization conditions. The polarization conditions were mimicked by placing uniform distributions of point charges behind the Ag111 slabs resulting in negative (cathode) and positive (anode) surface charge densities on the electrolyte facing surfaces.

The CMD simulations of the extended electrocatalyst system representing the electrochemical cell revealed the formation of a dense EDL at the cathode as the polarization was increased (Fig. 2). The density of water oscillates within 1 nm of the cathode surface indicating the formation of ordered layers of solvation, while it was found to be constant at 1 kg m^−3^ in the bulk phase. The simulations show a deeper penetration of water molecules into the outer Helmholtz plane (OHP; indicated by the dashed line at 1 Å from the electrode) of the cathode compared to the anode. K^+^ ions accumulated near the cathode while Cl^−^ anions accumulated at the anode and their respective concentrations in the EDL region increased with increasing surface polarization. This resulted in the compaction of the EDL and associated depletion of the CO_2_ near the electrocatalyst surface. The latter is fully covered by solvated alkali cations, which can be further considered as the reactive sites for CO_2_ activation instead of the bare metal surface.

### Outer-sphere CO_2_RR

**Fig. 3:**
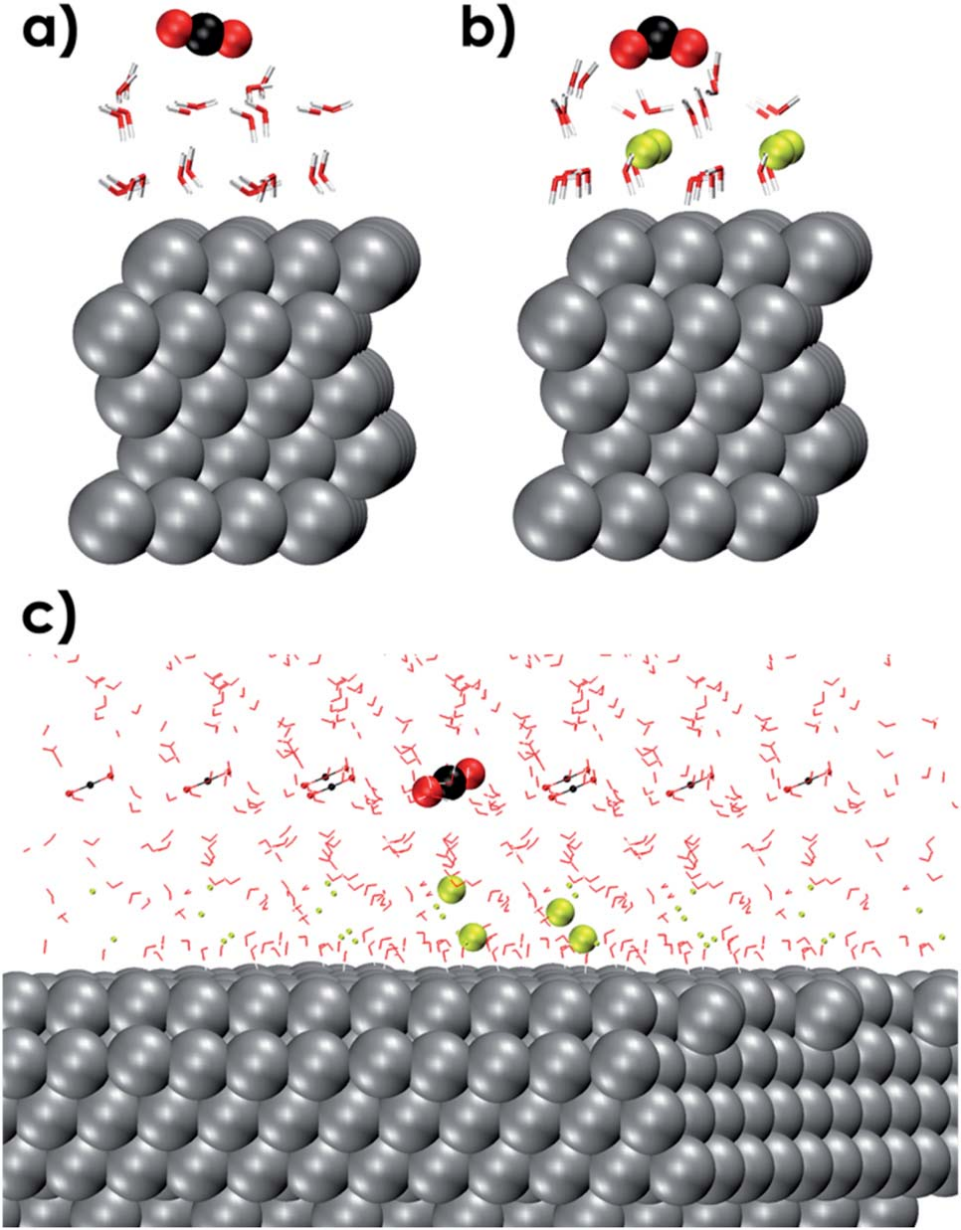
DFT optimized molecular models of a 4 × 4 × 5 Ag111 slab with one CO_2_ molecule in vacuum over the (a) Ag111-water interface (b) Ag111-EDL interface EDL = (Na^+^)_4_(H_2_O)_24_ and (c) fully solvated and periodic (in *x*, *y* and *z* directions) system used for AIMD simulations composed of a 4 × 4 × 5 Ag111 slab, 4Na^+^ cations and 61 water molecules. Periodic images have also been shown. The CO_2_ and Na^+^ species in the original simulation cell are shown as larger VdW spheres. Color code: C (black), Ag (silver), O (red), Na (Yellow), and H (white).

The outer-sphere reduction of CO_2_ over the solvated Ag111 surface was next investigated using periodic density functional theory (DFT) calculations (see the ESI for details†). The reactive events were simulated using a smaller molecular model representing the reaction environment near the Ag111 cathode (4 × 4 × 5 slab model). The initial static DFT calculations on the simplified models revealed the critical role of the EDL in the outer-sphere charge transfer eliciting indirect reduction of CO_2_. Indeed, the interaction of CO_2_ with an aqueous solvation layer on Ag111 is very weak and does not lead to notable perturbations of the adsorbed molecules (Ag111–(H_2_O)_24_–CO_2_, Fig. 3a). The situation drastically changes upon the introduction of sodium ions and the formation of the EDL (Ag111–(Na^+^)_4_-(H_2_O)_24_–CO_2_, Fig. 3b), which facilitates the reduction of CO_2_. The CO_2_ molecule in this case adopts a bent configuration due to the partial charge transfer from the silver slab. The bent anionic CO_2_ moiety is stabilized by hydrogen bonding with the neighbouring H_2_O molecules.

**Fig. 4:**
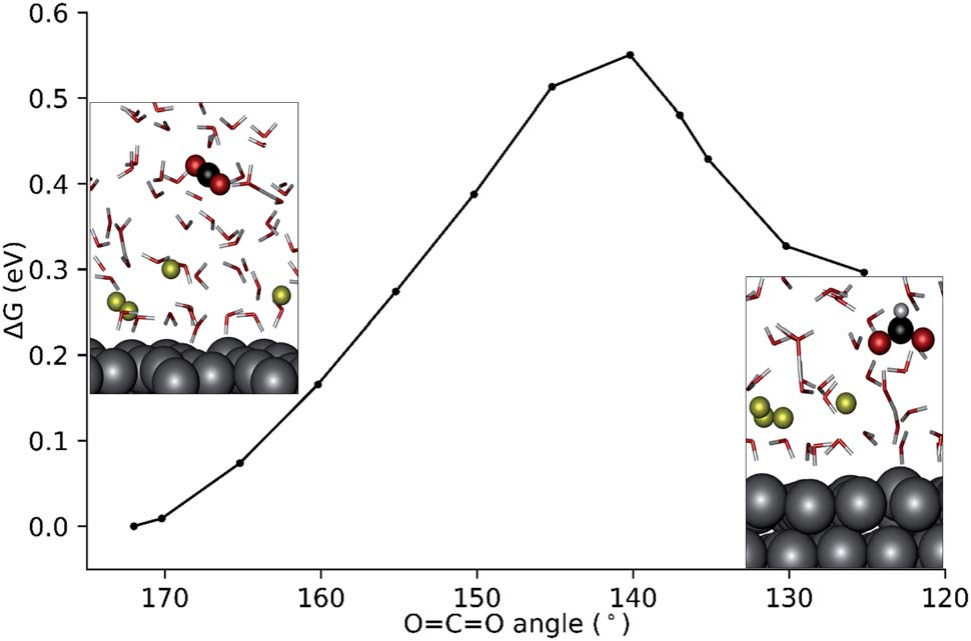
Computed Gibbs free energy profile for the homogeneous reduction of CO_2_ to formate using the ∠O=C=O angle as the reaction coordinate.

To better investigate the outer-sphere ET and the subsequent conversions of CO_2_ over the EDL, an extended fully solvated model was employed containing (Ag111–4Na^+^–(H_2_O)_61_–CO_2_, Fig. 2c) in combination with AIMD simulations (BLYP-PW-400 eV, VASP 5.5.4, for further details see the ESI†). The reactive environment was simulated with a 19 ps long AIMD simulation of CO_2_ in the solvated phase over the Ag111-EDL interface. CO_2_ was found to preferentially stay in the 3^rd^ and 4^th^ water layers of solvation at about 10 Å from the Ag111 surface. The average O=C=O angle was ∼172° during the runs. Two of the four Na^+^ cations forming the EDL were found at about ∼3 Å from the surface while the other two Na^+^ cations were located further away at ∼5 Å on the Ag111 surface (Fig. 3c).^43^ The water molecules within the EDL close to the surface facet were found to show a limited mobility. They pointed their protons towards the metal surface during the simulations. In the absence of the EDL, the water molecules preferentially oriented with O moieties pointing towards the Ag111 surface (Fig. 2a). Next, constrained AIMD simulations were carried out on 11 intermediate states representing different stages of the outer-sphere ET CO_2_RR. The O=C=O angle was chosen as the reaction coordinate (*Q*), and it was varied from 172° to 125°. The resulting Gibbs free energy profile along with the representative snapshots of the relevant reactant and product configurations are presented in Fig. 4.

The constrained AIMD simulations revealed that upon bending, the CO_2_ moiety diffused closer to the EDL. The transition state was located between O=C=O angles of 140°–145° (Fig. 4). Releasing the constraint at ∠O=C=O = 140° directly results in the formation of the formate product. The activation free energy barrier for the outer-sphere CO_2_RR is 0.55 eV with reference to the linear CO_2_ molecule.

Bader charge analysis at *Q* = 140° (equilibrated supercell at ∼18 ps) revealed a net atomic charge of −0.80 on the CO_2_ moiety. This is comparable to the Bader net atomic charge of −0.74 units computed for the 1e^−^ reduced CO_2_ radical in water (see the ESI†). Therefore, the bent CO_2_ moiety at 140° represents a 1e^−^ reduced CO_2_ radical. Upon further bending the transient radical species accepts an H^+^ from the solvent simultaneously with the second ET to yield the formate product. A snapshot of the proton transfer at ∠O=C=O = 137° is shown in the ESI.† Three water molecules coordinated to a Na^+^ in the EDL are actively involved during the proton transfer *via* H-bonding interactions. The bent CO_2_ moiety is strongly solvated forming 5 H-bonds.

**Fig. 5:**
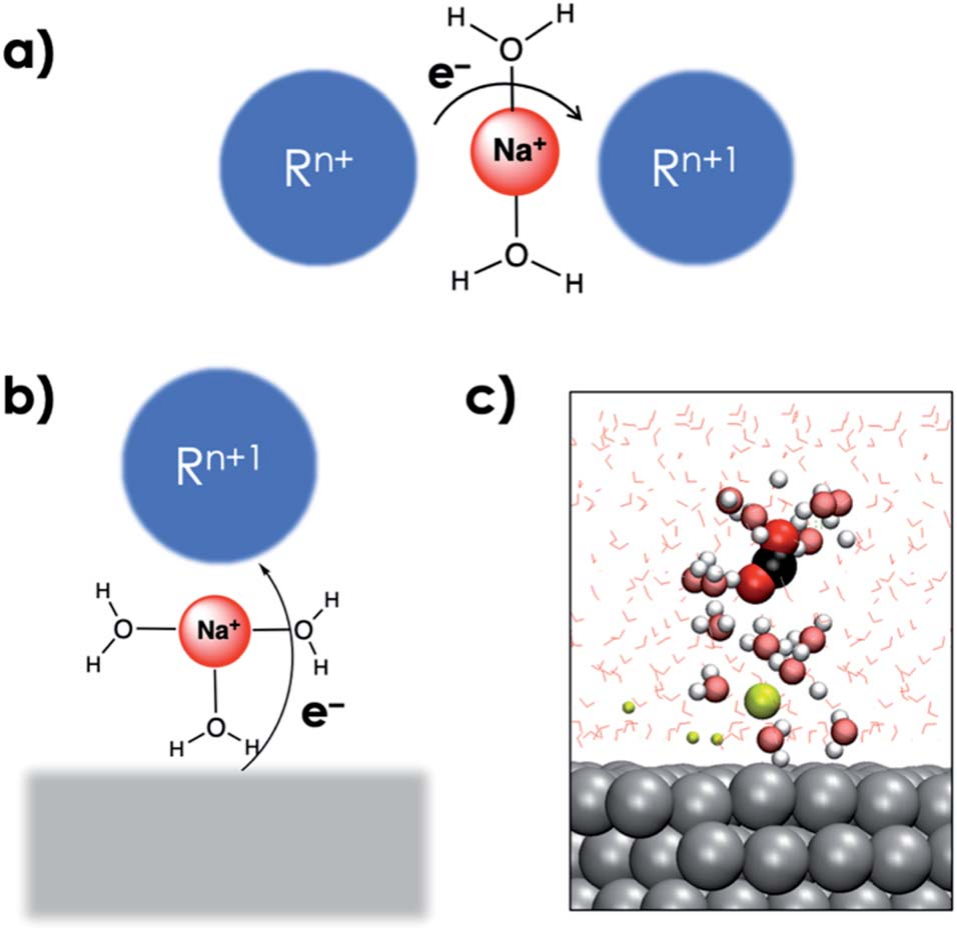
(a) Schematic representation of alkali cation promoted outer-sphere ET between two species in the homogenous phase. (b) Proposed schematic representation of alkali cation mediated outer-sphere ET from a cathode surface to a species in the homogeneous phase. (c) Snapshot of an AIMD trajectory with the O=C=O angle constrained at 140° showing the interaction between a solvated cation in the EDL and the solvated CO_2_ moiety analogous to the schematic depiction in (b). VdW representation is used for the CO_2_ moiety interacting with a solvated Na^+^ cation along with its first solvation shells which are shown in brushed metal colors. Other Na^+^ cations are shown as smaller spheres, and water molecules are shown *via* line representation. Periodic images have also been included.

The interaction with the EDL is critical for the reduction of CO_2_ and can be compared with cation mediated outer-sphere ET among species in the homogeneous phase (Fig. 5a).^44^ In the homogeneous phase solvated alkali cations have been reported to mediate outer-sphere ET between two species.^44^ AIMD simulations show that the solvated cations in the EDL can facilitate a similar outer-sphere ET between the cathode surface and CO_2_ (Fig. 5b and c). The critical role of the EDL in facilitating the CO_2_RR was further highlighted by the slow-growth approach (SGA) simulations that slowly bent the CO_2_ moiety when it was located far away from the EDL. In the absence of interaction of CO_2_ with the EDL, bending the CO_2_ moiety resulted in the formation of the HCO_3_^−^ species by the nucleophilic attack of water suggesting the importance of the EDL in facilitating electroreduction (see the ESI†).

The formate species formed *via* the outer sphere ET mechanism can undergo dehydration to form CO, which is expected to be favoured by low pH conditions near the EDL.^45^ Dehydration of formic acid/formate in acidic conditions is well-established chemistry. Consistently, AIMD simulations revealed similar free energy barriers for the dehydration of formate to CO in the solvated phase in the presence of the EDL (Ag111–4Na^+^–(H_2_O)_61_–CO_2_ system; 1.19 eV) and without the EDL (Ag111–(H_2_O)_61_–CO_2_ system; 1.26 eV) (see the ESI†). The presence of the EDL provides a small stabilization (0.07 eV) to the dehydration TS. The dehydration of formate to CO is expected to be more favourable with larger cations such as K^+^ and Cs^+^ where the pH near the EDL is lower.^45^

Experimental results for the CO_2_RR over Ag show that at high cathodic bias the hydrogen evolution reaction outcompetes the formation of CO. Jaramillo and co-workers showed that the partial current density for the formation of H_2_ exceeds that for CO at cathodic potentials below −1.3 V (RHE).^46^ The rate of formation of CO peaks around −1.1 V (RHE) and then decreases as the potential is lowered. Further analysis revealed that the decrease in CO formation was due to mass transport limitations. Both formate and CO require CO_2_ to reach the cathode surface for the CO_2_RR to proceed *via* an inner-sphere ET mechanism. Therefore, the rate of formate production is also expected to decrease around the same potential where CO production dips due to mass transport limitations. Contrastingly, the partial current density of formate, although always lower than CO and H_2_ kept growing as the cathodic bias was decreased.

An outer-sphere ET mechanism, which does not require mass transport of CO_2_ to the surface, can explain the increasing partial formate current density. At moderate to low cathodic bias, CO_2_ can reach the surface, and its adsorption is stabilized by the EDL^14,15^ leading to the production of CO (kinetically favoured) and HCOO^−^ (less favoured). Thus, the current density for CO and HCOO^−^ both increase as the applied voltage is lowered. At high cathodic bias the current density switches from kinetic control to mass transport limitations leading to decreased CO production. We suggest that the mass transport limitations (at least partially) result from a condensed EDL rather than only solubility and diffusion of CO_2_ in the electrolyte. CO_2_ is therefore available in the region close to the EDL and gets reduced to formate *via* an outer-sphere ET mechanism, which explains the increasing formate current density.

To gain further insight into HCOO^−^*versus* CO production we compare the free energy barriers for CO formation (inner-sphere ET) reported in the literature *versus* HCOO^−^ formation (outer-sphere ET) as computed by us. Based on the results reported by Chen and co-workers for the CO_2_RR over the Ag111 surface in the presence of a model EDL, a free energy barrier of 0.52 eV can be estimated for surface-mediated CO formation at an applied external potential of −1 V (SHE) at pH = 7.^14^ The current AIMD-computed free energy barrier of 0.55 eV at −1.45 V (SHE) (∼1 V *versus* the PZC)^47^ reflects a relatively higher barrier for the CO_2_RR *via* the outer sphere ET, explaining the lower partial current density for formate.

**Scheme 1:**
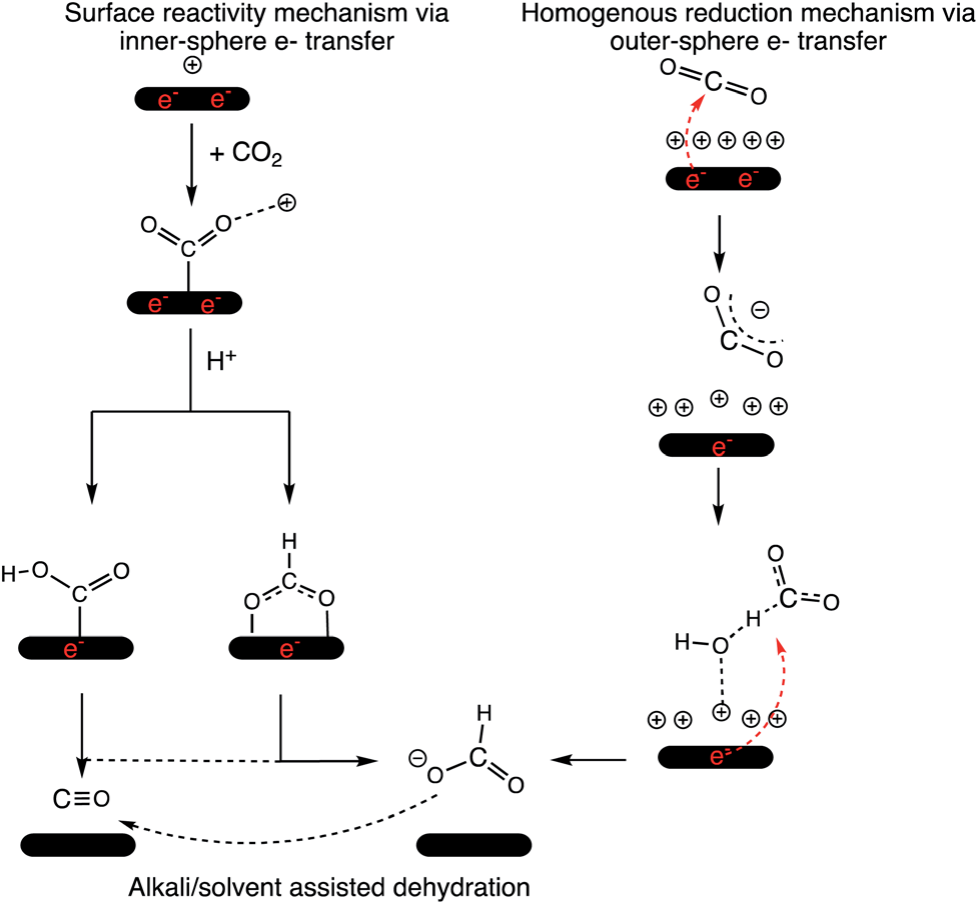
Mechanism of the CO_2_RR over a cathode surface *via* inner- and outer-sphere electron transfer. 2e^−^ that originate from the cathode surface and reduce the CO_2_ moiety are shown in red for representative purposes. The cathode surface is maintained at a constant potential in the electrolyzer.

Therefore, we propose that the mechanism of 2e^−^ reduction of CO_2_ is dependent on the applied bias. Scheme 1 summarizes the outer- and inner-sphere 2e^−^ mechanisms to produce CO and formate *via* the CO_2_RR. Jaramillo and co-workers also demonstrated the formation of >2e^−^ reduction products of the CO_2_RR over Ag at high overpotentials^46^ and an outer-sphere ET could potentially be involved in those mechanistic steps as well.

## Conclusions

We have explored the CO_2_RR over the Ag111 surface *via* an outer-sphere ET mechanism. Following a multiscale operando modelling strategy, we first simulated the multi-component electrolyte–cathode interface under various applied potentials. Investigation of density profiles of water, ions and CO_2_ revealed the formation of a condensed EDL within 1 nm of the cathode surface composed of cations and ordered layers of solvation at high overpotentials. This finding motivated the development of a smaller periodic model of the cathode-electrolyte interface which was used to investigate the reactive events during the CO_2_RR at high cathodic potentials *via* AIMD simulations. AIMD simulations showed that an outer-sphere ET mechanism resulted in the formation of formate species over the EDL. The formate species was further shown to undergo alkali promoted dehydration to CO with a moderate free energy barrier of 1.19 eV. The presence of the EDL was found to be the key to promote an outer-sphere ET CO_2_RR mechanism.

The outer-sphere ET CO_2_RR is a plausible mechanism to produce formate and CO under high cathodic bias. A surface-based, alkali promoted CO_2_RR is likely still the dominant mechanism for the formation of CO. Our calculations show that an alternative reaction channel to reduce CO_2_ is accessible in the presence of a dense EDL, and the reaction mechanism is a complex network of voltage dependent inner- and outer-sphere ET steps. Outer-sphere mechanisms should be further explored for heterogeneous electrocatalytic systems and can be especially relevant for electrocatalytic reduction of organic substrates which occur under high voltage conditions.

## Data availability

Data related to this publication is available *via* the 4TU database under the DOI: 10.4121/19142303.

## Author contributions

EAP conceived and supervised the project. VS carried out DFT and AIMD simulations. EK performed CMD simulations. All the authors discussed the results and wrote the manuscript.

## Conflicts of interest

There are no conflicts to declare.

## Supplementary Material

SC-013-D1SC07119J-s001
