# Supplementary material for: Solvent-mediated outer-sphere CO2 electro-reduction mechanism over the Ag111 surface
Source: Chem Sci. 2022 Feb 24;13(13):3803–8. doi: 10.1039/d1sc07119j (PMC8966634; doi:10.1039/d1sc07119j)
Supplement: SC-013-D1SC07119J-s001 [file SC-013-D1SC07119J-s001.pdf]

Electronic Supplementary Material (ESI) for Chemical Science.
This journal is © The Royal Society of Chemistry 2022

Supporting Information

Exploration of an outer-sphere CO_2 electro-reduction mechanism over Ag111 surface

Vivek Sinha,*[a] Elena Khramenkova[a] and Evgeny A. Pidko*[a]

[a] *Inorganic Systems Engineering Group, Department of Chemical Engineering, Delft University of Technology, Van der Maasweg 9, 2629 HZ Delft, The Netherlands*

Corresponding authors: Vivek Sinha (V.Sinha@tudelft.nl) and Evgeny A. Pidko (E.A.Pidko@tudelft.nl)

Table of Contents

S1. Computational methods: **2**

S2. Analysis of Bader Net Atomic Charges **6**

S3. Estimation of potential at the cathode **7**

S4. Bicarbonate formation **8**

S5. Formate formation during homogeneous CO_2 reduction **8**

S6. Bicarbonate formation in the presence of EDL 9

S7. Snapshot of proton transfer forming $HCOO^-$ 10

S8. PBE versus BLYP **10**

S9. EDL assisted thermochemical Dehydration of formate to CO **11**

S9.1 Reaction coordinate 11

S9.2 Gibbs Free Energy Profile 11

S10. Data availability. **12**

References **12**

Supporting Information

S1. Computational methods:

CMD model: CMD simulations were performed using the Large-scale Atomic/Molecular Massively Parallel Simulator (LAMMPS) (version from 22Aug 2018).[1] An aqueous solution of KCl that is a standard electrolyte in electrocatalytic studies, was confined between two charged Ag walls that represented the cathodic and anodic surfaces. The silver slabs with an electrolyte bulk, that consisted of K^+, Cl^- and CO_2 and H_2O molecules were constructed using Atomic Simulation Environment (ASE). The concentrations were set to correspond to the experimentally relevant conditions, namely, 0.86M for K^+, 0.86M for Cl^- and 0.06M for CO_2. The simulations were carried out using a supercell of 33.1x37.2x265.5 Å. The supercell was set to be periodic in the x and y directions and non-periodic in the z-direction. A vacuum spacing of 1.7 Å was set behind the silver slabs. The charging of the electrode surfaces due to externally applied potential was mimicked by introducing negative and positive ghost charges behind the Ag slabs. The calculated magnitudes of the electric field on the cathodic surface due to polarization induced from the ghost charges were 0 V/nm (no ghost charges), 0.05 V/nm, and 0.5 V/nm. The interatomic interactions between the components of the medium were modelled via Lennard-Jones (LJ) potential with the cut-off of 9.0 Å. The cross-terms were obtained using the Lorentz-Berthelot mixing rules. Water was modelled using the parameters of the SPC/E model,[2] that is widely used in simulations of the ions in aqueous solutions.[2b,c,d] The LJ parameters for CO_2, K^+, and Cl^- were taken from the literature.[3,4] The long-range interactions were calculated with the particle-particle-particle-mesh (PPPM) method. The O-H bonds and H-O-H angles were kept rigid within the SHAKE algorithm, whereas the C-O bonds and O-C-O angles were kept rigid via harmonic bond approximation. The temperature of the simulations was maintained using the Nose-Hoover thermostat at the value of 300 K. [3,4] We used a time step of 1 fs. The production runs were 90 ns long, excluding 10 ps of equilibration time in the beginning. The snapshots of trajectory for the further analysis were taken every 10 ps. The post-processing analysis was conducted using the MDAnalysis[20] and maicos_delft (https://gitlab.com/mdopke/maicos_delft) packages. For all the simulations, the system was initially equilibrated in the isothermal regime with different integration time steps (10^{-5}, 10^{-4}, 10^{-3}, 10^{-2}, 10^{-1} and 1) for 10^3 steps within each time step. However, the snapshots of atom quantities in the final dcd trajectory were included every 10 ps timesteps, giving the first 10 ps as equilibration time. Figure-SI 1 (left) shows that the energy of the production runs starting from the 10 ps is stabilized, with some small fluctuations present.

Supporting Information

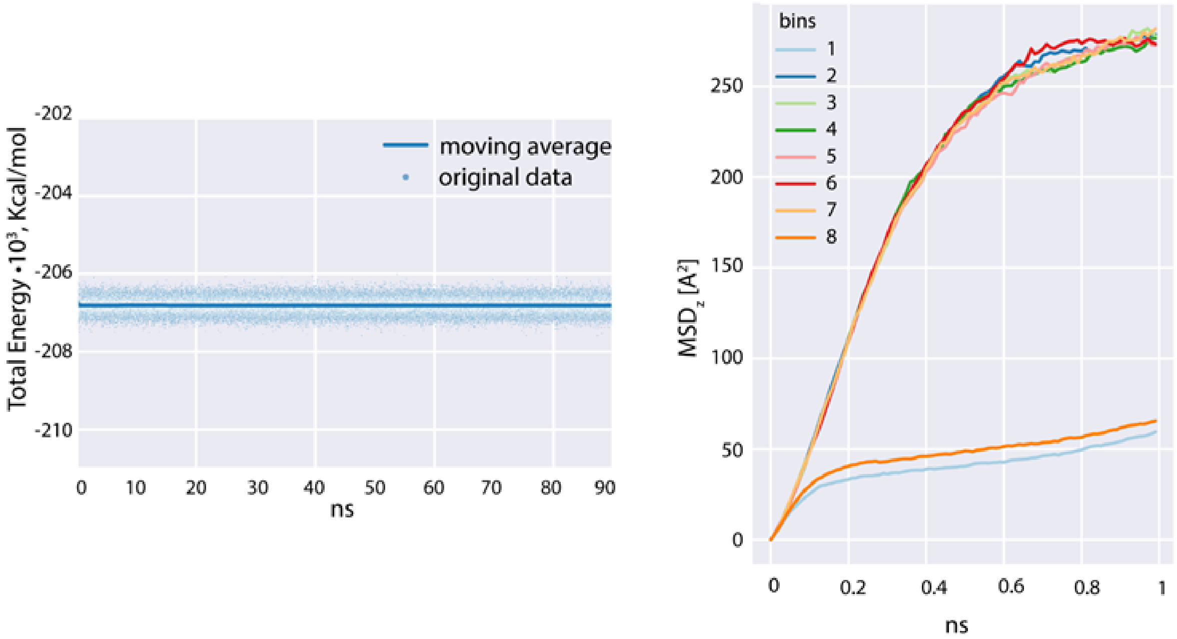

Figure-SI 1.(left) The total energy of the system with no electrif-field applied during the production run (original data in the scatter plot and the moving average is reflected in the line plot to account for the fluctuations). (right) Mean-squared displacement (MSD) of water in 8 bins along the z-direction in the system with no electric field applied. The first and the last bins (1 and 8) represent the area next to electrodes, whereas the other bins represent bulk diffusivity.

To calculate the self-diffusion coefficient of water, we have calculated the mean-squared displacement (MSD). The slope of the linear regression fit of MSD versus simulation time is proportional to the diffusion coefficient. For calculating the ensemble average, we have considered all the water molecules and multiple time origins. To indicate the difference in self-diffusivity in the bulk and next to the anode/cathode, the model was divided into 8 bins.

In Figure-SI 1 (right), we show the MSD versus time plot of water molecules along the z dimension – the longest in the model. The time frame between 20 – 400 ps was used for linear regression of the MSD_z versus time. As the first and the last bins were located near the electrodes (1 and 8), the self-diffusion coefficient were calculated for the central bins (2 – 7) which represent bulk diffusivity of water. The calculated diffusion coefficient based on the linear regressions resulted in a mean value of 2.78 $\bullet 10^{-9}$ m^2/s with the standard error of the mean equal to 0.01 $\bullet 10^{-9}$ m^2/s which compares well with the experimental value of $2.3 \bullet 10^{-9}$ m^2/s.[21a] , and CMD simulations of pure water using the SPC/E model at 298 K (in the range of $2.6 – 3.1 \bullet 10^{-9}$ m^2/s depedneing on the integrator time-step).[21b,c]

Table-SI 1 . Short-range pair interaction potentials used in the molecular dynamics simulations.

		ε	σ			ε	σ
Ag	Ag	4.56076	2.6325	Cl	H	0.0	2.2

Ag	C	0.50352	2.69475	Cl	K	0.1	3.8655
Ag	Cl	0.67533	3.51625	Cl	O	0.12464	3.783
Ag	H	0.00000	1.31625	Cl	Oc	0.12645	3.7165
Ag	K	0.67533	2.98175	H	H	0.0	0.0
Ag	O	0.84173	2.89925	H	K	0.0	1.6655
Ag	Oc	0.85394	2.83275	H	O	0.0	1.58300
C	C	0.05593	2.757	H	Oc	0.0	1.5165
C	Cl	0.07479	3.5785	K	K	0.1	3.331
C	H	0.00000	1.3785	K	O	0.12464	3.2485
C	K	0.07479	3.044	K	Oc	0.12645	3.182
C	O	0.09321	2.9615	O	O	0.15535	3.16600
C	Oc	0.09457	2.895	O	Oc	0.15760	3.0995
Cl	Cl	0.1	4.40	Oc	Oc	0.15989	3.033

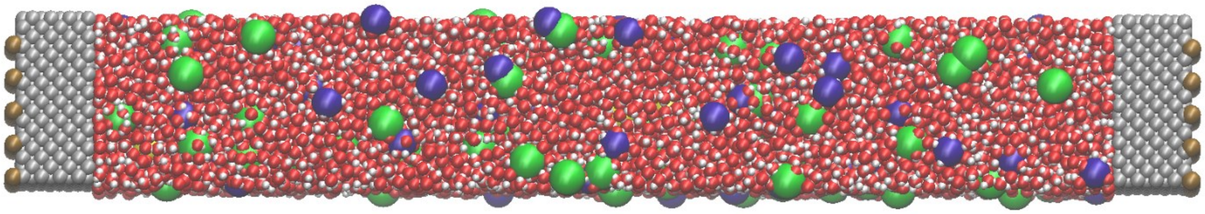

Figure-SI 3. The snapshot of the KCl electrolyte with CO_2 confined between two silver slabs simulated at zero potential. The surface on the left represents the anode, with positive ghost charges imposed behind the wall, while the right surface represents the cathode, with negative ghost charges imposed behind the wall. The color code is following: silver is grey, oxygen is red, hydrogen is white, potassium is violet, chlorine is green, carbon of CO_2 is yellow, the ghost atoms are light brown.

The electric field above the silver cathode was imposed by adding the ghost atoms behind the cathode and anode and was calculated according to Gauss's law that relates the distribution of the electric charge to the resulting electric field. The electric flux can be expressed as:

$\Phi_E = \frac{\Theta}{\varepsilon_{water} \cdot \varepsilon_{vacuum}}$, where Θ is the electric charge enclosed and ε_{water} is the dielectric constant of water and ε_{vacuum} is the dielectric constant of the vacuum. The values of the total induced charge were calculated according to the following:

$$\Theta = 25 \cdot e \cdot q$$

where q is the charge assigned to each ghost atom in LAMMPS simulation (for units style *real*), e is the electron charge equal to $1.6 \cdot 10^{-19}$ C and 25 is the number of ghost atoms imposed behind each surface. We have assumed that Ag is a perfect conductor and there is no charge density trapped in the bulk volume of Ag. We have also assumed that the dielectric constant of water does not change in the EDL.

The magnitude of the electric field passing through the cathode:

E = Φ_E/S, where S is the surface area of the cathode, which is assumed to be a rectangular prism.

Supporting Information

DFT calculations: we constructed a smaller molecular model of the electrocatalyst system consisting of a 4x4x5 Ag111 slab and performed density functional theory (DFT) calculations for the reactive events. The EDL was composed of 4 Na^+ cations per supercell which corresponds to ~ -1.0 V vs pzc (*vide infra*). The supercell was charge neutral. We performed geometry optimizations using periodic DFT calculations using the PBE[5] functional and projector augmented wave (PAW) potentials[19] with the the valence electrons expanded as plane waves (cutoff 450 eV) via VASP 5.4.4 suit of software.[6] In all DFT based geometry optimization was chose a fcc supercell of dimensions 11.53715 Å, 11.53715 Å, and 30.0 Å. The unit cell was constructed in the following manner: first 5 layers of 1x1 fcc cell of Ag111 was created with a lattice constant of 4.079 Å[18] and lattice vectors: (0.70710678, 0.0000000, 0.000000), (-0.35355339, 0.6123724, 0.000000), and (0.000000, 0.000000, 5.1961524). This 1x1 supercell was extended to create the 4x4x5 slab by changing the Bravais Matrix and adding additional atoms. The 4x4x5 Ag111 slab was fully optimized using DFT, and so were “4x4x5Ag111 slab+water molecules”, “4x4x5 Ag111 slab+water molecules + Na ions”, “4x4x5 Ag111 slab+water molecules + Na ions + CO2”, 4x4x5 Ag111 slab+water molecules + CO2”. Grimme’s D3 method with Becke–Johnson damping (D3(BJ)) was adopted for the van der Waals correction.[13,14] In the DFT based geometry optimizations we considered 24 water molecules for the solvation of the 4Na^+ cations over the 4x4x5 Ag111 slab. We further placed a CO_2 molecule at the EDL-vacuum interface and performed geometry optimizations. Calculations without Na^+ cations, that is Ag111-$(H_2O)_{24}$-CO_2 system were also performed where CO_2 was found to adopt a linear configuration upon geometry optimization. For the Ag111-$(Na^+)_4$-$(H_2O)_{24}$-CO_2 system, DFT based geometry optimizations resulted in CO_2 adopting a bent configuration and involved in hydrogen bonding with protons from H_2O moieties.

AIMD simulations: We further added 37 additional water molecules to solvate the entire supercell optimized via DFT. This system comprising of Ag111-4Na^+-$(H_2O)_{61}$-CO_2 was used for AIMD simulations in an NVT ensemble at 360 K using the Nose-Hoover thermostat. The BLYP exchange correlation functional was used for AIMD simulations. This combination of the XC functional and simulation temperature provides better description of the structure and dynamics of water, and the lower computational cost.[7–12] We reran a part of the constrained AIMD simulations using the PBE functional which resulted in similar barrier as obtained with the BLYP functional *(vide infra).* Grimme’s D3 method with Becke–Johnson damping (D3(BJ)) was adopted for the van der Waals correction.[13,14] The INCAR files used in our DFT and AIMD simulations are provided with the SI and can be referred to for further details on the simulation parameters. To generate a first-guess of the Gibbs free energy profile slow-growth approach (SGA) simulations were performed to traverse the Gibbs free energy surface from the reactant to the product state via the transition state using a reaction coordinate (Q) defined via a collective variable (bond lengths; bond angle etc.).[15–17] To further refine the Gibbs free energy profile, several intermediate values of Q were chosen and subjected to long (> 10 ps) constrained (fixed value of Q) AIMD simulations. In all AIMD simulations, constrained and unconstrained, we used an integrator time step of 1 fs. The average force (F) required to maintain the constraint was computed on an equilibrated trajectory via the bluemoon sampling method. The convergence of the force required to maintain the constraint was visually checked by plotting the force profiles for the last 5 ps of the simulation trajectory for each constrained AIMD run. The computed force profiles have been provided with the computational data provided via the 4TU database. The average force calculated on the last 1 ps of the equilibrated trajectory was integrated from the reactant (Q_i) to product (Q_f) state to obtain the corresponding Gibbs free energy change (ΔG): $\Delta G_{Q_i \rightarrow Q_f} = \int_{Q_i}^{Q_f} F \cdot dQ$.

S2. Analysis of Bader Net Atomic Charges

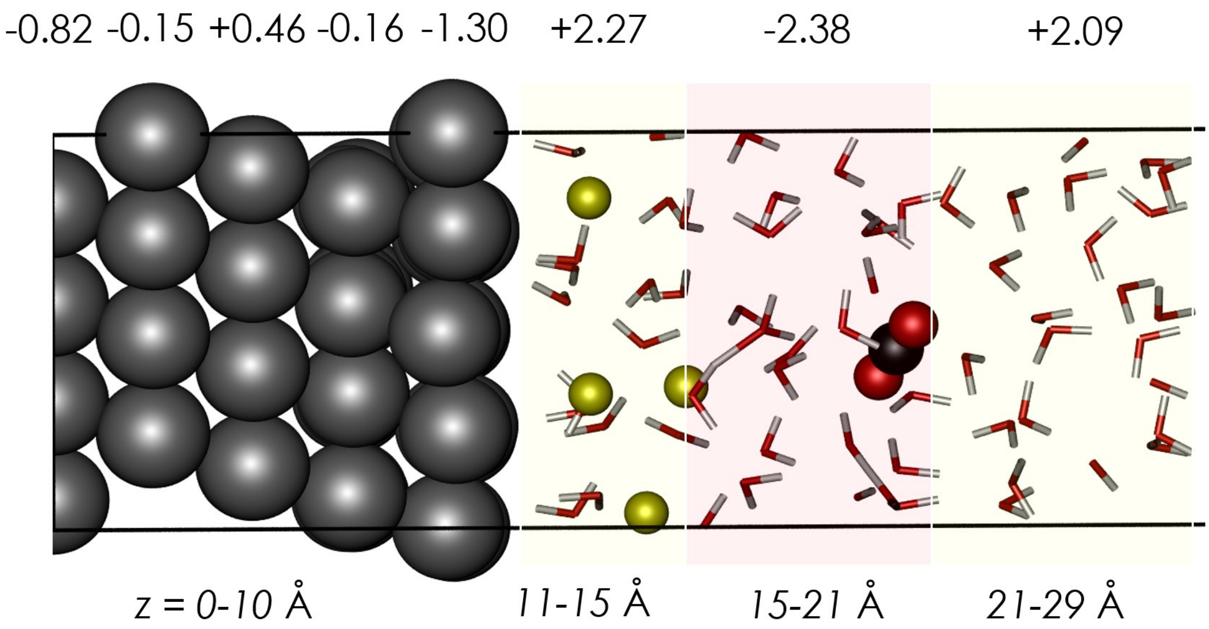

Figure-SI- 4. Analysis of Bader net atomic charges for a well equilibrated (19 ps) frame of CO_2 in the solvated phase over EDL-Ag111. The Bader net atomic charges for the five layers of Ag have been shown. The total Bader net atomic charges for various sections in the electrolyte phase have also been shown. The z-coordinate runs from 0 – 30 Å along the length of the simulation cell with 0-10 Å being the Ag111 slab.

S3. Estimation of potential at the cathode

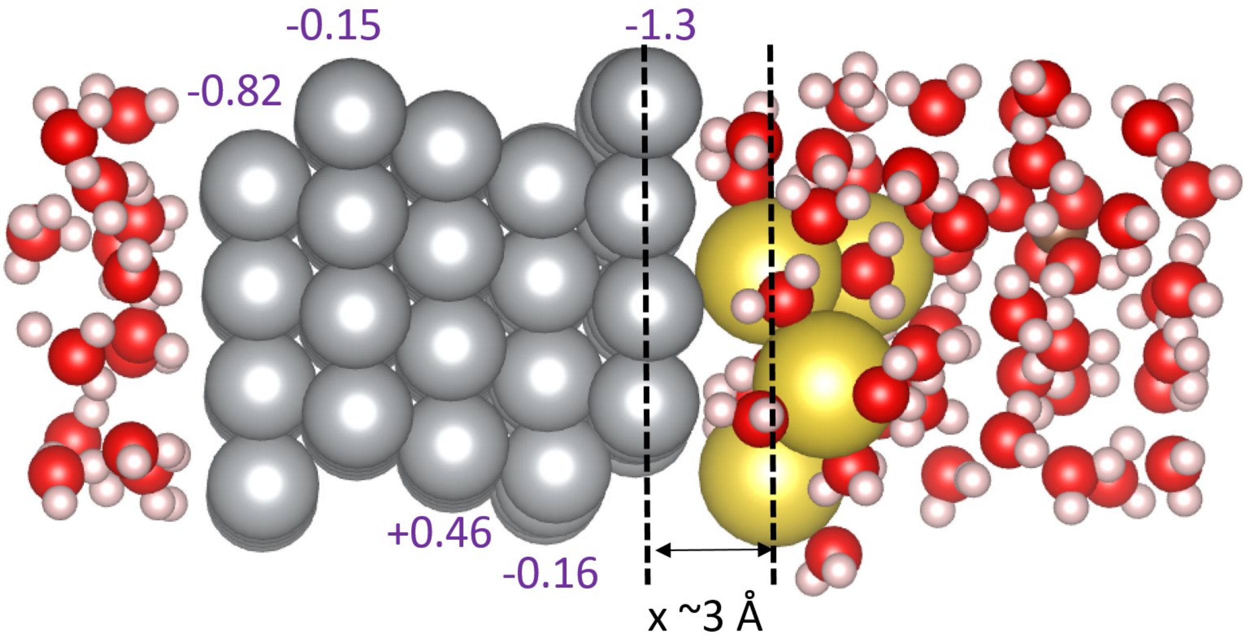

Figure-SI- 5. Ag111-EDL system. Total Bader net atomic charges are mentioned for each Ag layer.

$$\phi = \frac{\sigma}{C_H} + \phi_{pzc}$$
$$A = 115.27\ \text{Å}^2$$
$$\sigma = q * (-1.6) * \frac{10^{-19}\ (C)}{115.27 * 10^{-16}\ (cm^2)}$$
$$\sigma \sim\ -13.88q\ \mu C\ cm^{-2}$$
$$q = 1.3\ \text{(Bader net atomic charge on the Ag surface layer)}$$
$$\sigma = -18.04\ \mu C\ cm^{-2}$$
$$C_H = \frac{\epsilon\epsilon_0}{x}; x = \text{distance between Na}^+ \text{ and Ag surface} \sim 3\ \text{Å}$$
$$\epsilon = \text{relative permittivity of water} = 6^1$$

$$\epsilon_0 = \text{ permittivity of vacuum} = 8.85\text{x}10^{-12}\,\frac{\text{F}}{\text{m}}$$

$$C_H = \frac{6 * 8.85 * 10^{-12}}{3 * 10^{-10}} F\ m^{-2} \sim 18\ \mu F\ cm^{-2}$$
$$\phi - \ \phi_{pzc} = -13.88 * \frac{q}{C_H}\ \text{V (SHE)}$$

Therefore, C_H is estimated to be 18 μF cm^{-2} assuming that the relative permittivity of water in the EDL is at the minimum value which is a limiting case. We therefore also consider C_H = 25 μF cm^{-2} and

[1] We have taken the minimum relative permittivity of water in the EDL corresponding to a saturated electrolyte condition. This is different from the bulk value of 80.

$50\ \mu F\ cm^{-2}$ in our estimation of the surface potential. q which corresponds to the total surface charge on the reactive face can be taken as -1.3 (total Bader net atomic charge on the top layer) or -1.46 which is the total Bader net atomic charge on the top 2 layers. The estimated surface potentials are tabulated in Table-SI 2.

Table-SI 2. Calculated cathode potential at different value of q and C_H.

q	$\phi - \phi_{pzc}$ (V (SHE)) $with$ C_H = 18 $\mu F\ cm^{-2}$	$\phi - \phi_{pzc}$ (V (SHE)) $with$ C_H = 25 $\mu F\ cm^{-2}$	$\phi - \phi_{pzc}$ (V (SHE)) $with$ C_H = 50 $\mu F\ cm^{-2}$
-1.3	1.0	0.7	0.36
-1.46	1.1	0.8	0.41

S4. Bicarbonate formation

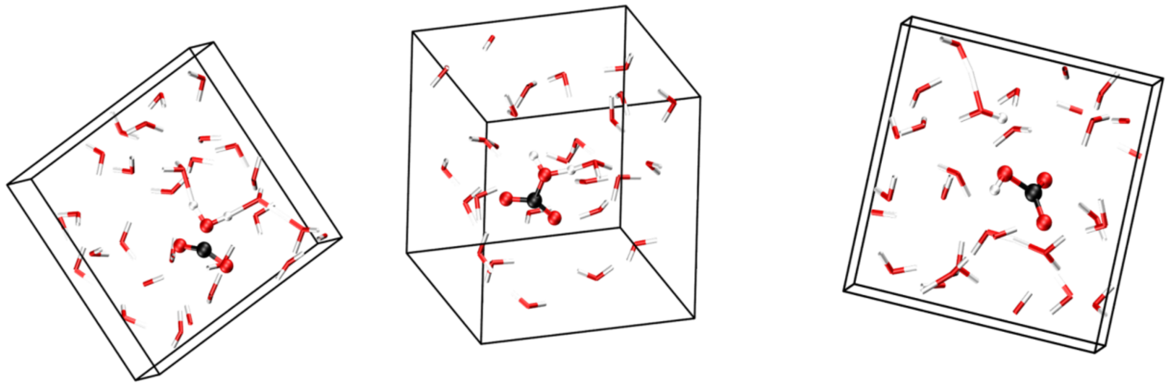

Figure-SI- 6. Snapshots from slow-growth approach simulations for formation of bicarbonate species from CO_2 solvated in a cubic cell with water. The O=C=O angle was used as the reaction coordinate.

S5. Formate formation during homogeneous CO_2 reduction

An outer-sphere ET would reduce CO_2 in the homogeneous phase. To gain insight into this *homogeneous* CO_2 reduction we carried out AIMD simulations of CO_2 in water (23 H_2O and 1 CO_2 per supercell). We performed three simulations: neutral, anionic, and dianionic. The neutral simulation consisted of a solvated CO_2 molecule in a neutral supercell. Introduction of 1e^- in the neural simulation resulted in the *anionic* system where CO_2 molecule was found to bend (<∠O=C=O> = 134.3°±3.1°). Both O moieties formed hydrogen bonds with water, and the C moiety was found to have a strong H-bonding interaction but did not get protonated during the simulations (Figure-SI 7a). Introduction of a second electron led to a doubly negatively charged *dianionic* system where formic acid quickly (within 50 fs of simulation time) formed (Figure-SI 7b,c). From the AIMD simulations of solvated CO_2 we conclude that an outer-sphere 2e^- transfer to CO_2 would drive the formation of formic acid/formate.

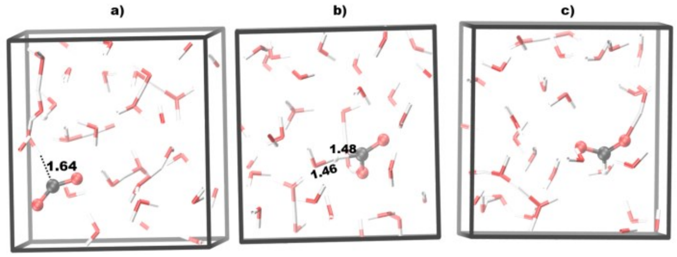

Figure-SI- 7.. Snapshots of AIMD trajectories: a) the anionic system where CO_2 adopts a bent configuration and is involved in H-bonding with water, b) proton being transferred from water to doubly reduced CO_2 in the dianionic system, and c) formate species formed in the dianionic system. All bond distances are show in Å units.

S6. Bicarbonate formation in the presence of EDL

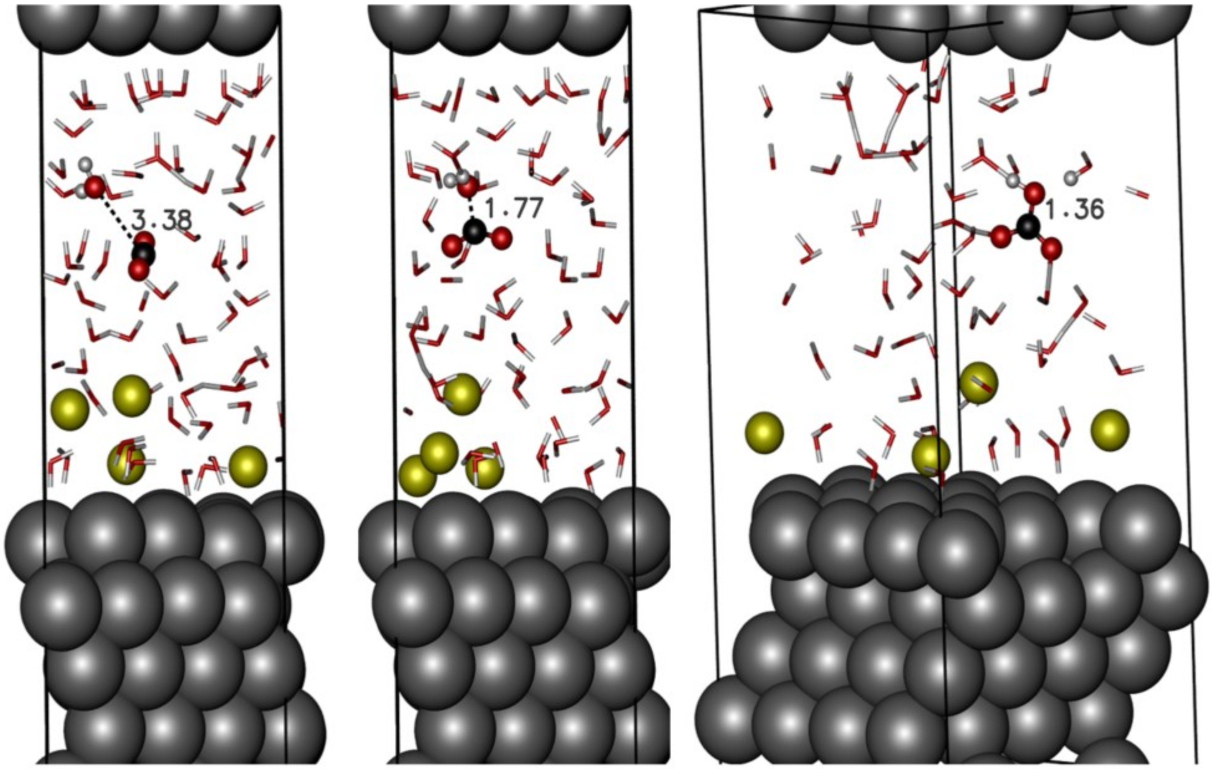

Figure-SI 8. Snapshots from slow-growth approach simulations showing the formation of HCO3- in Ag111-EDL supercell. The O=C=O bond angle was used as the reaction coordinate.

S7. Snapshot of proton transfer forming $HCOO^-$

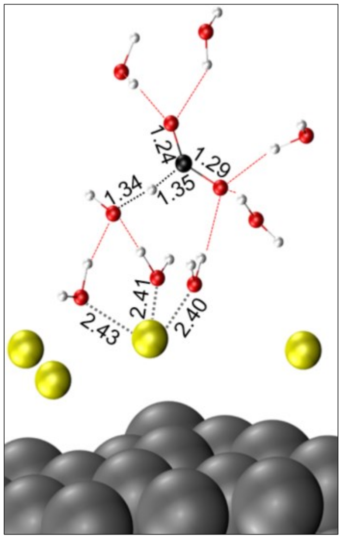

Figure-SI- 9. Snapshot from constrained AIMD simulations with O=C=O angle constrained at 137° showing proton transfer from water to a bent CO_2 moiety over the EDL. Only the most relevant water molecules have been shown. Bonds involved in the proton transfer are highlighted in dashed black and red (H bonds) lines. Color code: Na: Yellow; Ag: Silver; H: White; O: Red; C: Black.

S8. PBE versus BLYP

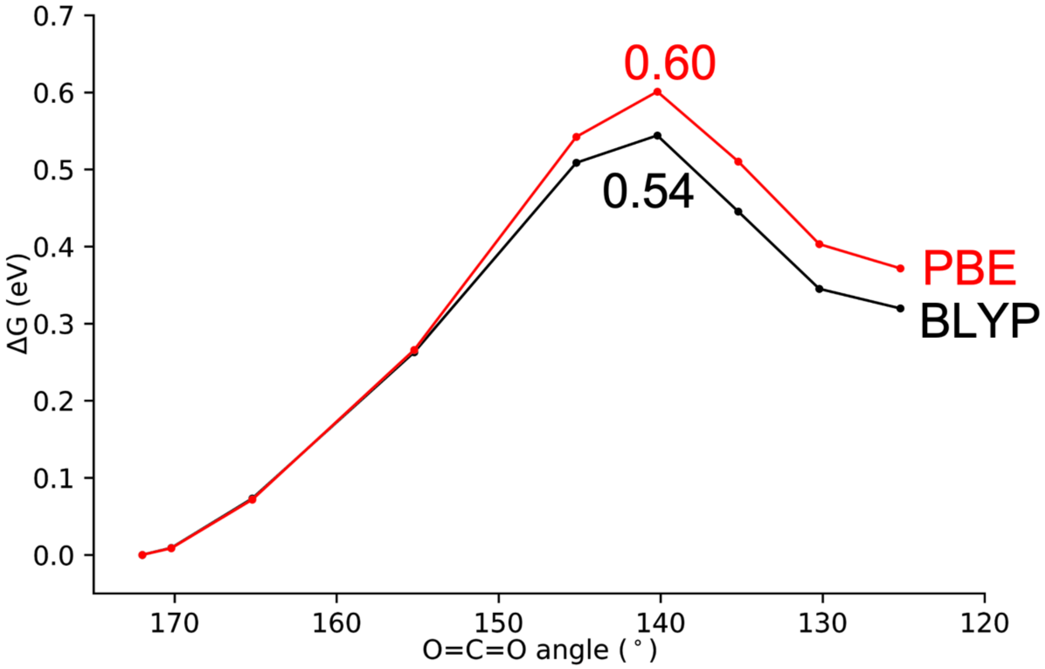

Figure-SI- 10. BLYP (black) and PBE (red) computed Gibbs free energy for reduction of CO2 to formate via constrained AIMD simulations. The respective free energy barriers are also mentioned. Fewer values of the reaction coordinate (Q) were used here compared to Figure 4 in the main text.

S9. EDL assisted thermochemical Dehydration of formate to CO

Using constrained AIMD simulations we also investigated the dehydration of formate to CO.

S9.1 Reaction coordinate

Reaction Coordinate and Gibbs Free Energy: formate dehydration

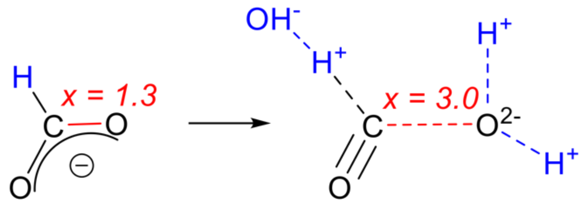

- Generate several configurations with different values of x
- Run simulations with constrained value of x until equilibration
- Force (F) is required to maintain the constrain, averaged (<F>) over trajectory for each x
- Compute $\Delta G = \int_{x=x_i}^{x=x_f} < F > dx$

Figure-SI- 11. Schematic description of the reaction coordinate and steps involved in the constrained AIMD simulations for dehydration of formate to CO.

S9.2 Gibbs Free Energy Profile

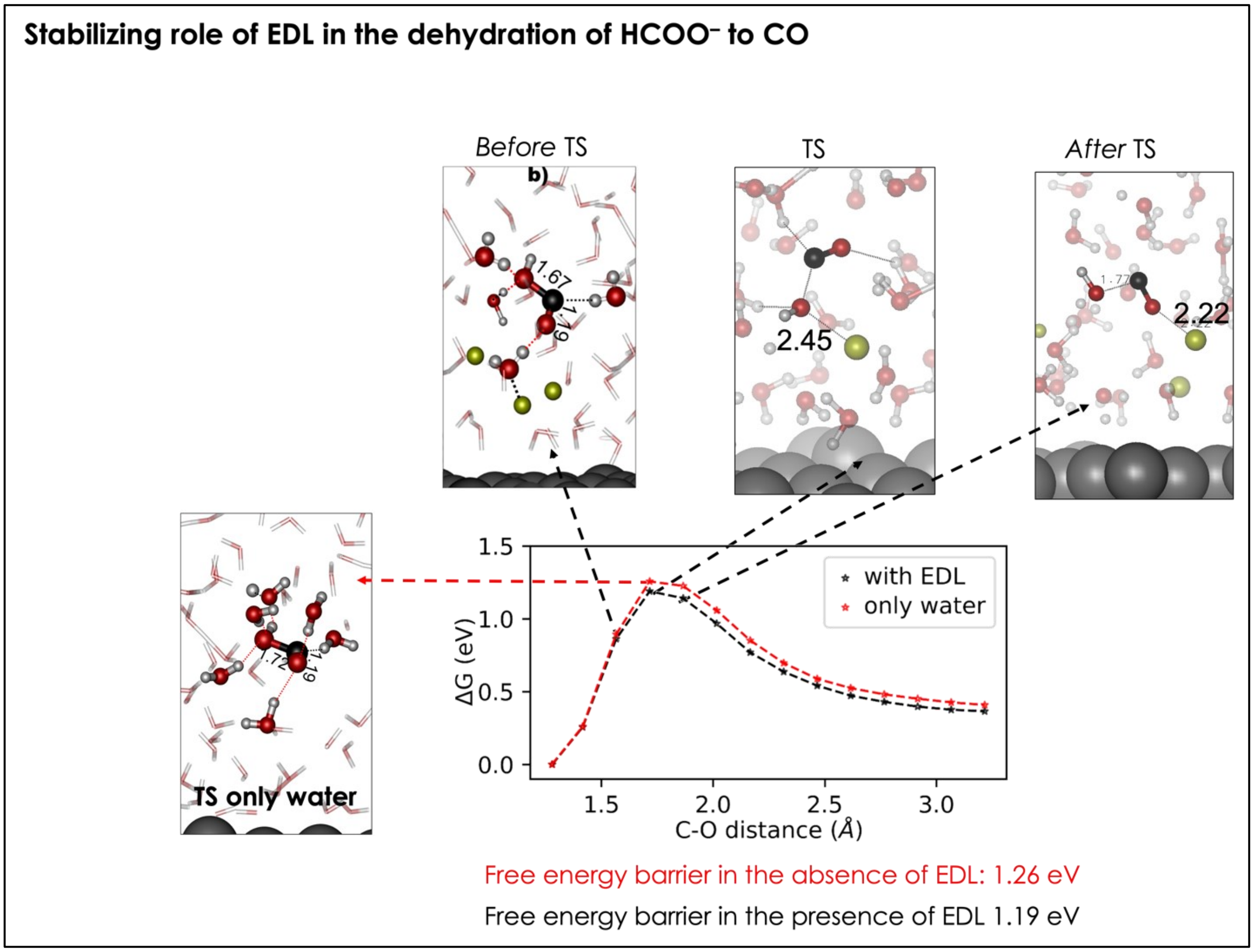

Figure-SI- 12. Computed Gibbs free energy profiles for the dehydration of formate assisted by the EDL (black) and unassisted (red). Snapshots of intermediates and TS have been included.

S10. Data availability.

Complete datasets including sample input files, optimized geometries, and trajectories from DFT, AIMD and CMD simulations including computed forced from constrained AIMD runs will be deposited to 4TU database and will be publically available under the DOI: 10.4121/19142303.

References

1 S. Plimpton, *J. Comp. Phys.*, 1995, **117**, 1–19.

2 a) H. J. C. Berendsen, J. R. Grigera and T. P. Straatsma, *J. Phys. Chem.*, 1987, **91**, 6269–6271. b) A. A. Dezfoli, M. A. Mehrabian and H. Hashemipour, *Chem. Engg. Comm.*, 2014, **202**, 1685–1692. c) Z. Mester and A. Z. Panagiotopoulos, *J. Chem. Phys.*, 2015, **143**, 044505. d) H. Heinz, R. A. Vaia, B. L. Farmer and R. R. Naik, *J. Phys. Chem. C*, 2008, **112**, 17281–17290.

3 S. Chowdhuri and A. Chandra, *The J. Chem. Phys.*, 2001, **115**, 3732–3741.

4 L. C. Nielsen, I. C. Bourg and G. Sposito, *Geochimica et Cosmochimica Acta*, 2012, **81**, 28–38.

5 J. P. Perdew, K. Burke and M. Ernzerhof, *Phys. Rev. Lett.*, 1996, **77**, 3865–3868.

6 G. Kresse and J. Furthmüller, *Phys. Rev. B*, 1996, **54**, 11169–11186.

7 M. Sprik, J. Hutter and M. Parrinello, *J. Chem. Phys.*, 1996, **105**, 1142–1152.

8 J. VandeVondele, F. Mohamed, M. Krack, J. Hutter, M. Sprik and M. Parrinello, *J. Chem. Phys.*, 2005, **122**, 014515.

9 B. G. Johnson, P. M. W. Gill and J. A. Pople, *J. Chem. Phys.*, 1993, **98**, 5612–5626.

10 C. Lee, W. Yang and R. G. Parr, *Phys. Rev. B*, 1988, **37**, 785–789.

11 A. D. Becke, *Phys. Rev. A*, 1988, **38**, 3098–3100.

12 V. Sinha, D. Sun, E. J. Meijer, T. J. H. Vlugt and A. Bieberle-Hütter, *Faraday Discuss.*, 2021, **229**, 89–107.

13 S. Grimme, J. Antony, S. Ehrlich and H. Krieg, *J. Chem. Phys.*, 2010, **132**, 154104.

14 S. Grimme, S. Ehrlich and L. Goerigk, *J. Comp. Chem.*, 2011, **32**, 1456–1465.

15 H. Oberhofer, C. Dellago and P. L. Geissler, *J. Phys. Chem. B*, 2005, **109**, 6902–6915.

16 T. K. Woo, P. M. Margl, P. E. Blöchl and T. Ziegler, *J. Phys. Chem. B*, 1997, **101**, 7877–7880.

17 C. Jarzynski, *Phys. Rev. Lett.*, 1997, **78**, 2690–2693.

18 W. P. Davey, *Phys. Rev.,* 1925, **25**, 753.

19 a) P. E. Blöchl, *Phys. Rev. B, 1994,* **50**, 17953–17979 b) G. Kresse and D. Joubert, *Phys. Rev. B, 1999,* **59**, 1758–1775.

20 N. Michaud-Agrawal, E. J. Denning, T. B. Woolf and O. Beckstein, *J. Comp. Chem.*, 2011, **32**, 2319–2327.

21 a) K. Krynicki, C. D. Green and D. W. Sawyer, *Faraday Discuss. Chem. Soc.*, 1978, ***66***, 199–208. b) O. A. Moultos, I. N. Tsimpanogiannis, A. Z. Panagiotopoulos and I. G. Economou, *Phys. Chem. B,* 2014, ***118***, 5532–5541c) D. Braun, S. Boresch and O. Steinhauser, *J. Chem. Phys.*, 2014, ***140***, 064107.
